# Bridging aging, immunity, and atherosclerosis: novel insights into senescence-related genes

**DOI:** 10.3389/fimmu.2025.1557266

**Published:** 2025-06-19

**Authors:** Yan Lu, Rong Yuan, Qiqi Xin, Keji Chen, Weihong Cong

**Affiliations:** ^1^ Laboratory of Cardiovascular Diseases, Xiyuan Hospital of China Academy of Chinese Medical Sciences, Beijing, China; ^2^ National Clinical Research Center for Chinese Medicine Cardiology, Xiyuan Hospital of Chinese Academy of Chinese Medical Sciences, Beijing, China

**Keywords:** atherosclerosis, biomarkers, vascular aging, macrophage, immunotherapy

## Abstract

**Background:**

Atherosclerosis (AS) is a chronic disease whose risk increases with age. Identifying reliable biomarkers and understanding the interactions between immune senescence and AS may provide important therapeutic opportunities for AS.

**Methods:**

The RNA sequencing and the single-cell RNA sequencing (scRNA-seq) dataset were downloaded from the Gene Expression Omnibus datasets, with all data derived from human tissues. Subsequently, differential expression analysis, weighted gene co-expression network analysis, accompanied by 3 machine learning algorithms, LASSO, SVM and RF, were performed to identify diagnostic genes. A nomogram and receiver operating characteristic analysis were used to assess diagnostic value. The immune cell infiltration and biological functions of the diagnostic genes were assessed by CIBERSORT and single-sample gene set enrichment analysis (ssGSEA). Next, we constructed a cellular map of AS plaques using scRNA-seq data. Senescence signatures in cell populations were quantified using the AUCell scoring algorithm. Intercellular crosstalk was explored using CellChat. Monocle2 was applied to elucidate macrophage developmental trajectories, exploring the relationship between biomarkers and immune cells. Finally, the expression of biomarkers and macrophage infiltration in aortic plaques of ApoE^−/−^ AS mice were evaluated using immunofluorescence.

**Results:**

A comprehensive screening identified 89 key senescence-related genes. Among these, *PDLIM1*, *PARP14* and *SEL1L3* were identified as biomarkers and showed high accuracy (AUC>0.7) in AS diagnosis. Based on ssGSEA, CIBERSORT and Pearson analyses, these biomarkers were found to correlate significantly with multiple immune cells, suggesting their potential involvement in immune infiltration processes. Pseudotime trajectory analysis revealed that PDLIM1, PARP14, and SEL1L3 exhibited stage-specific expression patterns during macrophage differentiation. Analyses based on CellChat indicated that senescent vascular cells predominantly communicate with macrophages, with differential expression of these biomarkers observed across distinct macrophage populations. Finally, PDLIM1 expression was downregulated and PARP14 and SEL1L3 expression was upregulated in the aortic root of AS mice. Macrophages showed significant accumulation in the aortic root of AS mice with a dysregulated M1/M2 macrophage ratio, which is consistent with the bioinformatics analysis.

**Conclusions:**

In conclusion, senescence-associated genes may drive macrophage transformation, and they show high potential for surveillance and risk stratification in AS, which may inform immunotherapy for AS.

## Graphical Abstract

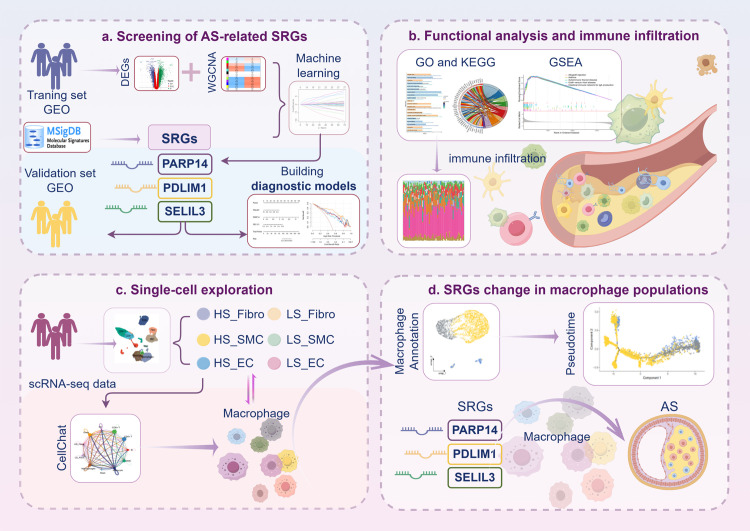

## 1 Introduction

Atherosclerosis (AS) is an age-related chronic inflammatory disease characterized by a progressive accumulation of plaques, which is the underlying pathology leading to myocardial infarction and stroke, and accounts for approximately 31% of global mortality (1). AS plaque formation is primarily driven by endothelial dysfunction, which promotes low-density lipoprotein (LDL) aggregation in the intima by compromising vascular barrier integrity. The modification of LDL, primarily oxidation LDL (oxLDL), promotes the recruitment and infiltration of monocytes into the vessel wall, leading to the accumulation of cholesterol-enriched foam cells that contribute to plaque growth and necrotic core formation (2, 3). Over time, the development of necrotic fragments, plaque destabilization, and subsequent rupture can result in fatal acute cardiovascular events. In recent years, personalized medicine has been recommended for the comprehensive management of AS because it uses strategies to predict individual susceptibility and target prevention to personalize healthcare (4). Currently, statins remain the cornerstone of AS treatment, effectively lowering LDL levels through the inhibition of *HMG-CoA* reductase. Although statins reduce the risk of major adverse cardiovascular events in clinical trials, there is still a substantial residual risk. In recent years, numerous researchers have attempted to combine immune and anti-inflammatory therapies and reduce major adverse cardiovascular events. Notably, anti-inflammatory treatment with canakinumab targeting the interleukin-1β (*IL-1β*) innate immune pathway led to a significant reduction in recurrent cardiovascular events (5, 6). In addition, studies have shown that immune checkpoint proteins and co-stimulatory molecules play an important role in the regulation of atherosclerosis (7–9). These studies provide new insights into the importance of immune modulation in AS.

Aging has been identified as a predominant risk factor for cardiovascular disease (CVD) (10). It affects the vasculature even before the development of AS. Generally, aging is associated with arterial wall remodeling, characterized by accelerated elastin network fragmentation and degradation, accompanied by abnormal collagen fibers proliferation. In addition, reduced endothelial nitric oxide synthase activity triggers decreased nitric oxide (NO) bioavailability, and disruption of the integrity of the endothelial barrier, all of which together contribute to increased arterial stiffness (11). Aging affects the immune system in complex ways, and various components of the immune system are involved in the formation of AS. A notable characteristic of aging is macrophage accumulation and altered polarization. Recent studies suggest that M1-like macrophages, along with senescent cells, may be a major source of pro-inflammatory cytokines (e.g., *IL-1β*, *IL-6*, and *TNF*) during aging (12). The downregulation of *CD36* and *CD163* expression on the surface of senescent macrophages significantly impaired recognition and phagocytosis of oxidized LDL and apoptotic cells (13, 14). Senescence drives chronic inflammatory progression in AS by remodeling monocyte/macrophage differentiation trajectories and phagocytosis (15). A study revealed that aged mice with chronic or acute hyperlipidemia exhibited a greater degree of macrophage infiltration into AS lesions than younger mice (16). In the context of AS, senescent cells contribute to the degeneration of the fibrous cap, which is critical for preventing plaque rupture. The clearance of these senescent cells through senolytics interventions has been shown to restore the numbers of vascular smooth muscle cells (VSMCs) and increase cap thickness. Given the progressive functional decline of the aging system, the incidence of acute cardiovascular events also increases substantially with age (17). Understanding the interactions among aging, adaptive immunity, and atherosclerosis is essential for addressing and mitigating cardiovascular complications related to aging. The aim of this study is to reveal underlying immune mechanisms associated with aging-related biomarkers in AS by integrating bioinformatics methods and machine learning strategies, combined with single-cell RNA sequencing (scRNA-seq) and functional validation experiments. Ultimately, this study aims to lay a theoretical foundation for personalized interventions in AS, which could inform the future development of precision therapies for AS patients.

## 2 Methods

### 2.1 Dataset collection

In this study, we obtained the original microarray datasets from the Gene Expression Omnibus (GEO) database (http://www.ncbi.nlm.nih.gov/geo, accessed on 4 September 2024), Specifically, we utilized the GSE43292 dataset from the GPL6244 platform and the GSE100927 dataset from the GPL17077 platform. The GSE43292 dataset comprises 32 carotid AS samples and 32 carotid non-AS samples (18). while the GSE100927 dataset includes 29 AS carotid plaques and 12 control carotid arteries without AS lesions (19). Additionally, the annotated files for GPL6244 and GPL17077 were downloaded from GEO. For genes with multiple probes IDs without prior filtering, we calculated the mean expression value of all probes to represent the expression level of individual genes. Furthermore, the GSE28829 dataset includes 13 early and 16 advanced human carotid AS plaque (20). The GSE120521 dataset consisted of 4 stable and 4 unstable AS plaque samples (21). GSE41571 dataset consisted of 6 stable and 5 unstable AS plaque samples (22). The 3 datasets previously mentioned were utilized as validation sets. In addition, we used the scRNA-seq dataset GSE159677 to conduct a more comprehensive analysis of the cell populations expressing key biomarkers (23). The samples in GSE159677 were derived from whole carotid AS plaques and matched adjacent non-AS tissues from the same patients. Before conducting the formal analysis, the data were normalized using the limma (v3.60.6) R package. Senescence-related genes (SRGs) were sourced from MSigDB 3.0 (https://www.gsea-msigdb.org/gsea/msigdb, accessed on 10 September 2024). All human data used in this study were obtained from public repositories. According to the original publication, the studies were approved by the local ethics committee and conducted in accordance with the Declaration of Helsinki.

### 2.2 Identification of differentially expressed genes

We identified differentially expressed genes (DEGs) from GSE43292 and GSE100927 using the Bioconductor R package limma. Differential expression analysis was performed using a linear model with empirical Bayesian variance adjustment. The parameters |Log2 fold change|>0.585 (about 1.5-fold change) and Benjamini-Hochberg adjusted *P*-value (FDR) < 0.05 were used as screening criteria for DEGs. In addition, volcano plots of DEGs were constructed using the ggVolcano R package.

### 2.3 Construction of weighted gene co-expression networks

In this study, the R package “WGCNA” was used to construct weighted gene co-expression network analysis (WGCNA). First, screening top 1000 highly variable genes performed hierarchical clustering on the study samples to detect and exclude abnormal samples. The function blockwiseModules() was used to construct the co-expression network with the following settings: minModuleSize=30 and mergeCutHeight=0.25. Subsequently, a soft-thresholding power of β = 16 was selected using the function pickSoftThreshold to construct the scale-free network. Following this, the adjacency matrix was built and converted to a topological overlap matrix (TOM), and the dissimilarity was used to build the gene dendrogram and module colors. Each module was designated with a distinct color identifier, and the module diagnostic genes represented the expression profile of the entire module. After selecting the candidate modules, we defined |Module Membership|>0.8 and |Gene Significance|>0.2 as the screening criteria for key genes in the candidate modules.

### 2.4 GO and KEGG enrichment analyses

To elucidate the underlying biological mechanisms through which overlapping DEGs regulate phenotypic changes, we inferred the molecular networks and cellular processes in which these genes may be involved by identifying the functional categories and signaling pathways in which they are significantly enriched. We analyzed the Gene Ontology (GO) and Kyoto Encyclopedia of Genes and Genomes (KEGG, updated October 2025) pathways for the target genes using the R package clusterProfiler (v4.12.6). The org.Hs.eg.db annotation package provided Homo sapiens-specific GO terms, such as biological processes (BP), cellular components (CC), and molecular functions (MF), alongside KEGG pathways. A corrected *P* < 0.05 was considered statistically significant. The Benjamini-Hochberg procedure was applied for multiple testing correction to control the false discovery rate (FDR).

### 2.5 Feature gene selection based on multiple machine learning methods

Machine learning prediction models include the least absolute shrinkage and selection operator (LASSO) algorithm, support vector machine (SVM) models, random forest (RF) models. The LASSO algorithm, a form of logistic regression, is employed to enhance predictive performance through variable selection and regularization (24). The LASSO logistic regression model (family = “binomial”) was implemented using the “cv.glmnet” function from the glmnet R package with default standardization of predictors (standardize = TRUE). The parameters were set with alpha = 1 and nlambda = 1,000, with lambda.min automatically selected by the 10-fold cross-validation implemented within the cv.glmnet function. The SVM algorithm finds the optimal variables as a supervised machine learning method to support vectors. It is a widely used supervised machine learning protocol for classification and regression, which recursively eliminates the least important features based on linear SVM-derived importance rankings to optimize variable selection. The “Caret” package of the grid search method is used to select hyperparameters for all classifiers through 5-fold cross-validation on the training dataset. The SVM has demonstrated its capability to identify the diagnostic significance of biomarkers with high discriminative power, a process facilitated by the “e1071” package (25). The RF analysis was performed using the “RandomForest” function. The optimal number of variables per split (mtry) was selected via grid search using the tuneRF function, and the ntree parameter defaults to 500 (17). The top 20 key genes were selected based on the feature weights mean decreased accuracy (MDA) and mean decrease Gini (MDG). Subsequently, overlapping genes were identified in the three machine learning methods for further analysis.

### 2.6 The construction of nomogram

In order to evaluate the comprehensive diagnostic performance of the characterized genes, a multivariable logistic regression model was first established using the “rms” package to predict the binary outcome of AS. The “rms” package was applied to construct a nomogram, and a calibration curve was established to evaluate the accuracy of the nomogram. In the nomogram, each gene is assigned a specific score, and the cumulative scores of the 3 genes are employed to predict the risk of AS. Finally, a decision curve analysis was performed using the “rmda” package to evaluate the net benefit of the nomogram prediction, and this analysis supports their potential value in guiding AS risk stratification decisions.

### 2.7 The ROC curve analysis and expression analysis

In the GSE43292 and GSE100927 datasets, we validated the accuracy of the screened diagnostic genes by performing receiver operating characteristic (ROC) curve analysis utilizing the “pROC” package. Each gene was individually assessed as a continuous predictor, and the area under the curve (AUC) was calculated to quantify its diagnostic performance. Genes with an AUC > 0.7 were deemed diagnostically valuable for the disease. To further validate the accuracy of the diagnostic genes, we performed independent ROC analyses on the validation datasets (GSE28829, GSE120521, and GSE41571) and compared their diagnostic performance with the GSE100927 and GSE43292 datasets. The expression levels of hub genes between AS and control samples were displayed in the boxplots generated by the “ggplot2” in R package.

### 2.8 Single-sample gene set enrichment analysis

The single-sample gene set enrichment analysis (ssGSEA) was used to identify the potential functions of diagnostic genes. The reference gene set was sourced from the MSigDB (C2 gene set). *P* < 0.05 (FDR-corrected) was used as the criterion for significant enrichment.

### 2.9 Correlation analysis between infiltrating immune cells and diagnostic genes

The immune cell infiltration was calculated using the web tool CIBERSORT (http://CIBERSORT.stanford.edu/, accessed on 20 September 2024) to explore the immune microenvironment of AS. The analysis employed the LM22 reference set, which comprises 22 human immune cell subtypes. The number of permutations sets was 1000. The *P*-value < 0.05 (FDR-corrected) in the CIBERSORT results was retained. The result of immune cell infiltration was visualized by ggplot2 package. Pearson correlation analysis was employed to determine the relationships between diagnostic genes and immune cells.

### 2.10 Single-cell analysis

We included AS core plaque and patient-matched proximal portion transcriptome data from GSE159677. The analysis was conducted utilizing the “Seurat” R package (v5.2.0). Firstly, quality control was performed to screen out cells that met the following criteria: a gene count per cell >200, and mitochondrial gene percentage <10%. Subsequently, data normalization was performed using the NormalizeData function. For downstream analysis, the “vst” method in the FindVariableFeatures function was used to select the top 2000 variably expressed genes. Principal component analysis (PCA), was performed on normalized data for dimensionality reduction, followed by unsupervised graph-based clustering using the FindClusters() function with a resolution parameter of 0.3. The “Harmony” package was used to remove batch effects across dissociated scRNA-seq raw data. Employing unsupervised cluster analysis and unified manifold approximation and projection (UMAP), discrete cell clusters were discerned within each scRNA-seq dataset. In the first round of cell annotation, cells were identified by the following markers: CD4^+^ T cells (*CD3D*, *IL7R*, *LTB*, *CD2*), CD8^+^ T cells (*CD8B*, *GZMK*, *CCL5*, *GZMA*), endothelial cells (ECs; *VWF*, *PECAM1*, *PLPP1*, *PLVAP*), fibroblasts (Fibro; *LUM*, *FGF7*, *DCN*, *COL1A1*), monocytes (*S100A9*, *S100A8*, *FCN1*, *LYZ*), macrophages (*SELENOP*, *C1QA*, *HLA-DPA1*, *CCL3*), dendritic cells (DC, *CD1C*, *CLEC10A*, *FCER1A*, *HLA-DQA1*) and B cells (*JCHAIN*, *IGHG1*,*CD69*, *IGKC*, *CD79A*). SMC (*TAGLN*, *MYH11*, *TPM2*), and mast cells (*TPSB2*, *TPSAB1*, *CPA3*, *MS4A2*) (23, 26). Subsequently, macrophages were extracted for further annotation, specifically C1Q^+^ macrophages (*C1QA*, *C1QB*, *C1QC*, *FOLR2*), TREM2^hi^ macrophages (*TREM2*, *SPP1*, *FTL*, *APOE*), FCN1^+^ macrophages (*FCN1*, *S100A9*, *S100A8*, *VCAN*) (27–29).

### 2.11 Pathway activity and cell–cell communication analysis

In the scRNA-seq dataset, Fibro, VSMC and ECs were categorized into high senescence (HS) and low senescence (LS) subgroups based on the senescence-associated transcriptional signature “FRIDMAN. SENESCENCE. UP”, obtained from the MSigDB, using the AUCell_exploreThresholds function. This method dynamically determines the optimal thresholds by fitting a bimodal distribution of AUCell (v1.26.0) scores. Cellchat (v1.6.1) was employed to analyze cell-cell communication using a normalized gene expression matrix. To investigate intercellular communication associated with senescence, we performed CellChat analysis of normalized gene expression matrices for HS and LS cell subtypes versus other cell types.

### 2.12 Single cell trajectory analysis

We used Monocle2 to study inferred developmental trajectories among macrophage subsets. Monocle2 uses reverse graph embedding to learn the sequence of gene expression changes that each cell undergoes in the dynamic biological samples provided. This approach enables the precise positioning of each cell along the trajectory of gene expression changes. Initially, the data normalized and clustered via the “Seurat” R package were loaded into a monocle object. Dimensionality reduction was performed using the “reduceDimension” command and the DDRTree algorithm. Subsequently, the trajectory was constructed using the “plot_cell_trajectory” command with default parameters. Cells were sorted along pseudotime trajectories using genes dynamically selected by Monocle2’s differentialGeneTest and dispersionTable. The root cells of the pseudotime trajectories were automatically identified using the orderCells() function. The dynamic trend of the expression levels of the diagnostic genes over pseudotime were depicted in individual graphs using the plot_genes_in_pseudotime function.

### 2.13 Animal models and aortic valve harvesting

Male C57BL/6J mice (n=4) and ApoE^-/-^ mice (n=4), all 8 weeks of age, were purchased from Beijing Vital River Laboratory Animal Technology Co., Ltd. (Beijing, China). All mice were given free access to food and water under constant temperature and humidity environmental conditions (12-hour light/dark cycle). To induce AS, four ApoE^-/-^ mice were switched from a normal food chow to a high-fat diet (comprising 21% fat, 0.15% cholesterol) for 24 weeks. The other four C57BL/6J mice were fed a normal food diet for 24 weeks and served as controls. At the end of the study, mice were anesthetized by intraperitoneal injection of sodium pentobarbital (60 mg/kg), after which the aortic valves were harvested, fixed in 4% paraformaldehyde and placed at 4°C for 24 hours for subsequent studies. The animals were cared for in accordance with the Guidelines for Care and Use of Laboratory Animals published by the US National Institutes of Health (NIH Publication, 8th Edition, 2011). All procedures involving experimental animals were approved by the Ethics Review Committee for Animal Experimentation of Xiyuan Hospital, China Academy of Chinese Medical Sciences (Approval No. 2024XLCO54-2).

### 2.14 Evaluation of AS lesions

For evaluation of AS lesions in the aortic sinus, the aortic root was dehydrated and embedded in paraffin. Serial 4-μm sections were obtained in the aortic sinus using a slicer (Leica RM2016, Wetzlar, German). Sections were stained with hematoxylin-eosin (HE, Sevicebio, Wuhan, China) to quantify plaque area. Tissue sections were subsequently scanned and photographed at 400× magnification under a digital scanner (3DHistech corporation, Budapest, Hungary) and quantitatively analyzed using Image J software (v1.53t; National Institutes of Health, Bethesda, MD, USA).

### 2.15 Immunofluorescence staining

Tissue sections of mice aortic valves underwent deparaffinization and hydration, followed by antigen retrieval through boiling in sodium citrate at 100°C for 30 min. The prepared tissue sections were closed with 5% BSA for 30 min at room temperature. For immunofluorescence staining, the samples were incubated overnight at 4°C with the following antibodies: PDLIM1 polyclonal antibody (1:1000; Cat# 11674-1-AP; Proteintech), PARP14 polyclonal antibody (1:7000; Cat# bs-19886R; Bioss), SEL1L3 polyclonal antibody (1:5000; Cat# bs-19626R; Bioss), F4/80 FITC-labeled polyclonal secondary antibody (1:2000; Cat# GB113373; Servicebio), CD80 polyclonal antibody (1:1000; Cat# GB114055; Servicebio), CD163 monoclonal antibody (1:3000; Cat# GB15340; Servicebio), CD36 polyclonal antibody (1:5000; Cat# GB112562; Servicebio). Tissue sections were incubated with secondary antibodies (Alexa Fluor 594 labeled goat anti-mouse IgG, HRP conjugated Goat Anti-Rabbit IgG, Cy5 conjugated Goat Anti-rabbit IgG or Cy3 conjugated Goat Anti-Rabbit IgG,1: 500; Servicebio, Wuhan, China) were incubated at 37 °C away from light for 50 min, washed 3 times with PBS, and then counterstained with 4′,6-diamidino-2-phenylindole (DAPI). Finally, images were acquired using a fluorescence microscope (Nikon Eclipse C1, Japan) and the immunofluorescence data were quantified as mean fluorescence intensity using ImageJ software.

### 2.16 Statistical analyses

All data were expressed as mean ± standard deviation. Comparison of data obeying a normal distribution was performed using a two-tailed independent samples t-test. *P*-value ≤ 0.05 was used to define significance.

## 3 Results

### 3.1 Identification of DEGs

**Figure 1:**
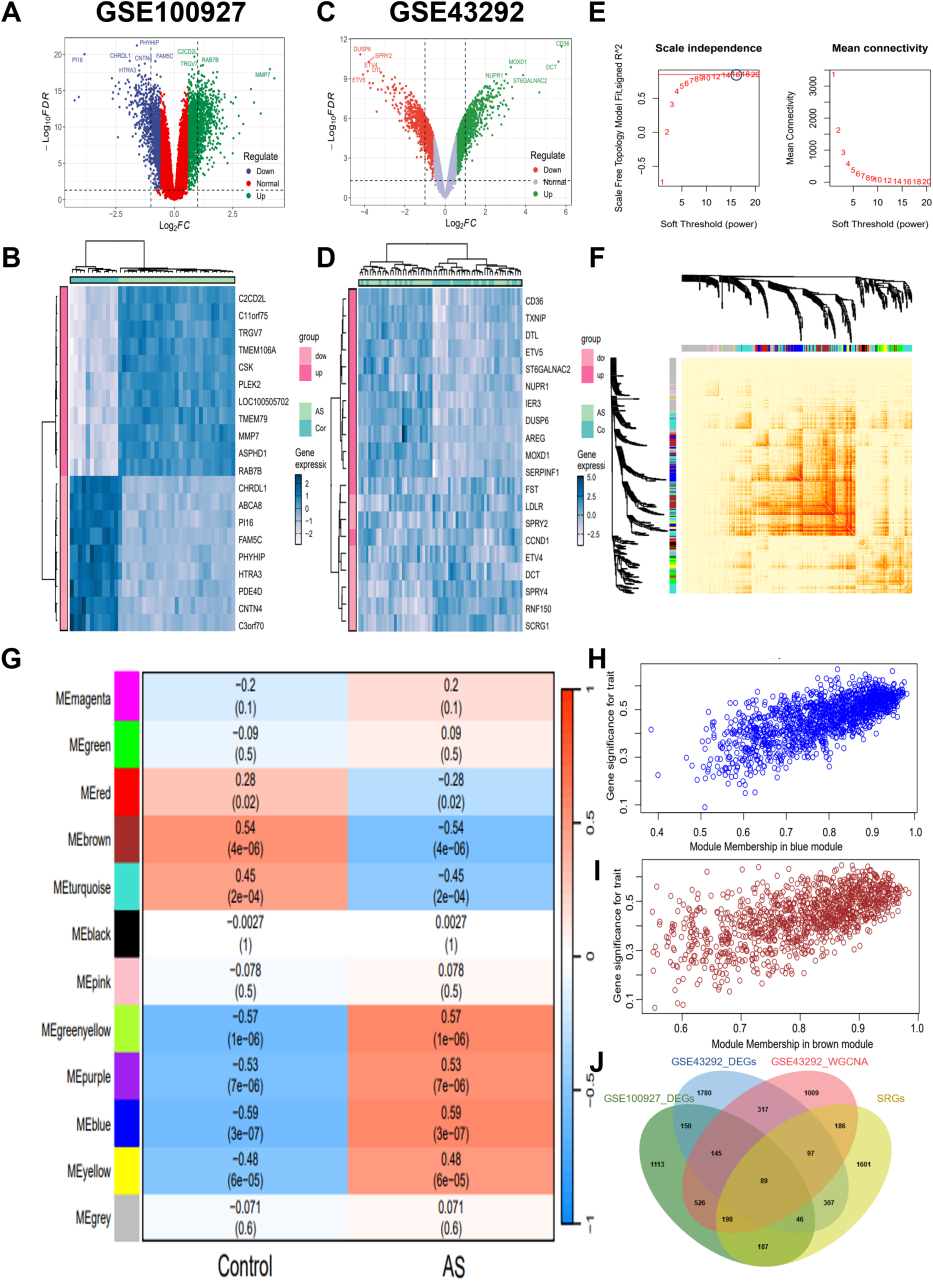
Screening for key genes. **(A, C)** Volcano map of DEGs distribution in GSE100927 and GSE43292. **(B, D)** Heatmap of DEGs in GSE100927 and GSE43292. The legend at the top right represents the log fold change in genes. The horizontal axis represents each sample and the vertical axis represents each gene. Blue and white colors represent low and high expression values, respectively. **(E)** Identification of the optimal β value by using scale-free topology model, and selection of β = 16 as the soft threshold based on average connectivity and scale-independence. **(F)** The network heatmap showing gene dendrogram and modular eigengenes. **(G)** The heatmap of correlation analysis of modular eigengenes with clinical status. in AS. The correlation (upper) and *P*-value (bottom) of module eigengenes and status of AS were presented. Red color indicates positive correlation and blue color indicates negative correlation. **(H)** The correlation plot of blue module affiliation with gene significance in blue module. **(I)** The correlation plot of brown module affiliation with gene significance in brown module. **(J)** Intersection of key module genes with DEGs was obtained by Venn diagram and a total of 234 AS key genes were identified. GSE100927 dataset: AS groups, *n*=29 biologically replicated experiments; control groups, *n*=12 biologically replicated experiments. GSE43292 dataset: *n*=32 biologically replicated experiments.

Initially, we explored the different gene expression patterns in the AS and control samples in the GSE43292 and GSE100927 datasets. The differential expression analysis revealed 2454 DEGs in GSE100927, including 1397 up-regulated and 1057 down-regulated genes (**Figures 1A, B**). Concurrently, a total of 2931 DEGs, including 1653 up-regulated and 1278 down-regulated genes, were identified in GSE43292 (**Figures 1C, D**). These DEGs were visualized in the volcano plots, and the heatmaps showed the top 20 DEGs, which were selected based on the log2FC.

### 3.2 Weighted co-expression network construction and core module selection

In order to further screen the key genes of AS, we performed WGCNA on gene expression data from the GSE43292 dataset. A soft threshold power of 16 was selected based on the scale-free topology criterion and mean connectivity analysis (**Figure 1E**). A total of 12 co-expression modules were identified using this power, representing groups of co-expressed genes with similar expression patterns. The hierarchical relationships among these modules are depicted in the cluster dendrogram shown in **Figure 1F**. Subsequently, a WGCNA-based module-trait correlation analysis was conducted to identify gene modules significantly associated with AS (**Figure 1G**). The blue module exhibited the highest positive correlation with AS, comprising 1416 genes (r=0.59, *P*=3e-07; **Figure 1H**), while the brown module demonstrated the highest negative correlation with AS, consisting of 1152 genes (r=-0.54, *P*=4e-06; **Figure 1I**). Accordingly, the blue and brown modules were selected as the focus modules for further analyses. In addition, we intersected the DEGs from the GSE43292 and GSE100927 datasets with the genes identified by WGCNA, resulting in a total of 234 key genes (**Figure 1J**).

### 3.3 GO and KEGG pathway analysis

**Figure 2:**
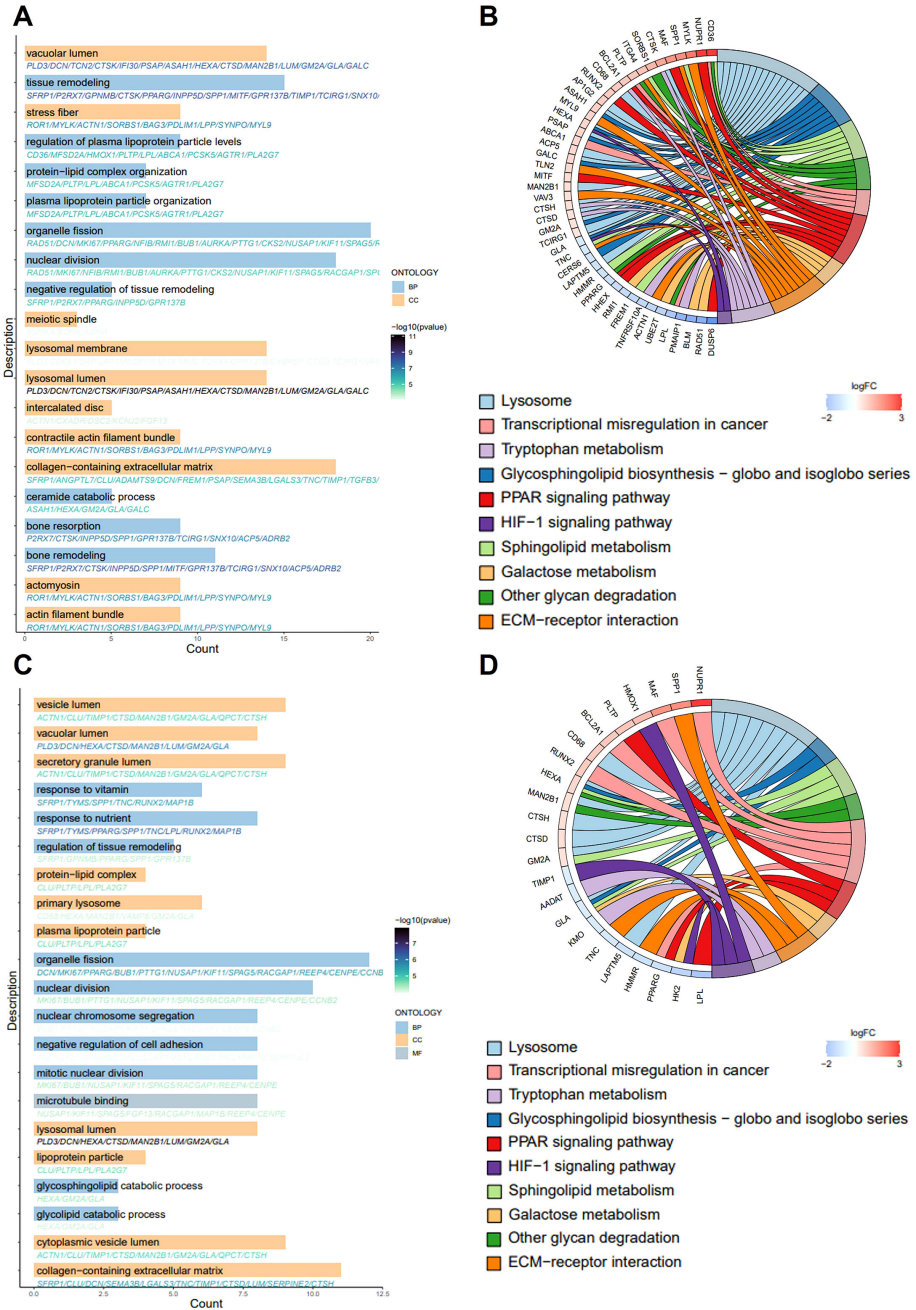
GO and KEGG pathway enrichment analysis. **(A, C)** The bar graph shows the GO-enriched terms: biological process (BP), cellular composition (CC) and molecular function (MF). The x-axis indicates the number of genes enriched to the entry, and thy-axis labels represent GO terms. **(B, D)** The chord plot shows KEGG-enriched items. The red color in the graph expresses gene upregulation and blue color indicates gene downregulation. Gene involvement in KEGG terms is identified by colored connecting lines.

Subsequently, we performed GO and KEGG functional enrichment analyses for the key genes. **Figure 2A** presents the significantly enriched GO terms. In the BP category, the key genes were primarily associated with the regulation of macrophage derived foam cell differentiation, macrophage cytokine production, foam cell differentiation, T cell homeostasis, neutrophil differentiation, B cell activation, and other immune and inflammatory processes. In CC analysis, the key genes were significantly involved in lysosomal lumen, vacuolar lumen, stress fiber, contractile actin filament bundle, collagen-containing extracellular matrix, plasma lipoprotein particle, among others. The KEGG enrichment analysis revealed significantly enrichment in pathways such as lysosome, sphingolipid metabolism, ECM-receptor interaction, PPAR signaling pathway, cholesterol metabolism, transcriptional misregulation in cancer and other pathway (**Figure 2B**). AS is recognized as an age-related disease, highlighting the importance of further elucidating its molecular pathogenesis from an aging perspective. We obtained SRGs from MSigDB and identified the intersection of AS key genes with SRGs using a Venn diagram, resulting in the identification of 89 key senescence-related genes (**Figure 1J**). GO analysis of these genes showed that key senescence-related genes were significantly enriched for BP, such as organelle fission, glycosphingolipid catabolic process, mitotic nuclear division, glycolipid catabolic process, inflammatory response to wounding and negative regulation of B cell activation. Among the CC enrichment results, significant enrichment was observed lysosomal lumen, vacuolar lumen, collagen-containing extracellular matrix, mitotic spindle, high-density lipoprotein particle, and contractile actin filament bundle, among others (**Figure 2C**). Subsequently, the key senescence-related genes were further analyzed using KEGG pathway functional enrichment analysis. This analysis identified significant enrichment in pathways such as lysosome, glycosphingolipid biosynthesis, PPAR signaling pathway, galactose metabolism, ECM-receptor interaction and tryptophan metabolism (**Figure 2D**).

### 3.4 Screening of hub genes with diagnostic value via machine learning

**Figure 3:**
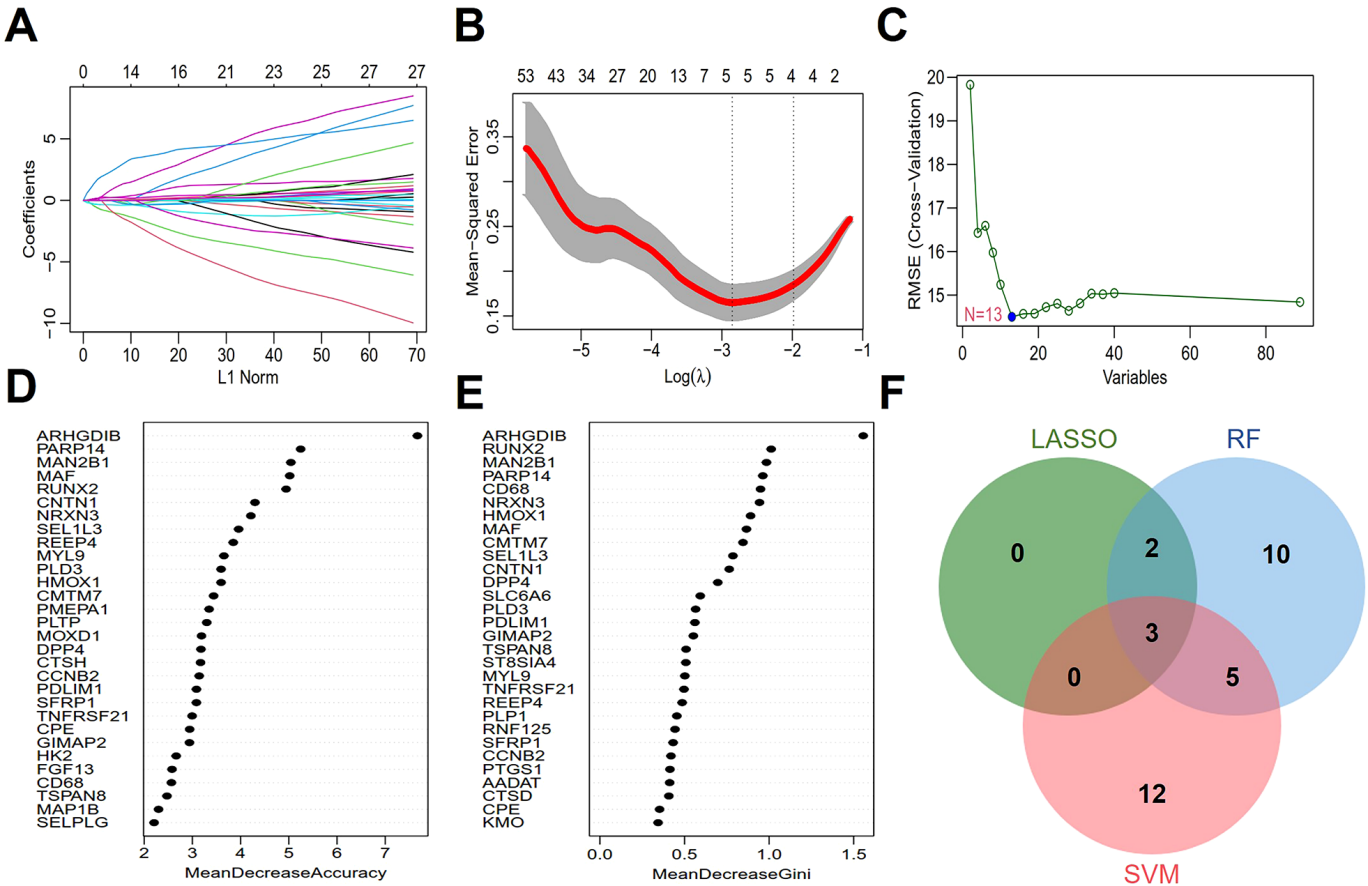
Screening of hub genes with diagnostic value via machine learning. **(A, B)** Feature selection in LASSO regression. **(C)** Expression validation of biomarker labeled genes selected by SVM. **(D, E)** Genes are arranged in descending order according to MeanDecreaseAccuracy and MeanDecreaseGini values in RF. **(F)** The Venn diagram depicting 3 common genes between LASSO, SVM, and RF that chant identified as aging-related diagnostic genes in AS.

Three machine learning algorithms, LASSO, SVM and RF, were used to identify AS diagnostic genes from 89 key senescence-related genes. LASSO logistic regression was employed to perform binary classification analysis for predicting disease subtypes. Gene coefficient trajectories and binomial deviance curves were visualized to evaluate feature selection stability (**Figures 3A, B**). The hub genes were selected from the variables corresponding to the optimal penalty parameter values. Ultimately, the LASSO regression identified five hub genes, including *PDLIM1*, *CNTN1*, *HMOX1*, *PARP14*, and *SEL1L3*. The SVM was optimized using a polynomial kernel for feature screening, based on optimal parameter selection (**Figure 3C**). The top 20 genes identified by the SVM as the most informative features included *CENPE*, *CCNB2*, *SEL1L3*, *ARHGDIB*, *PARP14*, *PDLIM1*, *GPR137B*, *CMTM7*, *TNFRSF21*, *VAMP8*, *DCN*, *NFIX*, *HMMR*, *LUM*, *CLU*, *TSPAN8*, *ST8SIA4*, *AADAT*, *PTTG1* and *SPAG5*. For the RF algorithm, the diagnostic errors were visualized, and the candidate genes were ranked in a descending order according to the importance of the variables (**Figures 3D, E**). The top 20 genes were identified as significant, including *ARHGDIB*, *RUNX2*, *MAN2B1*, *PARP14*, *CD68*, *NRXN3*, *HMOX1*, *MAF*, *CMTM7*, *SEL1L3*, *CNTN1*, *DPP4*, *SLC6A6*, *PLD3*, *PDLIM1*, *GIMAP2*, *TSPAN8*, *ST8SIA4*, *MYL9*, *TNFRSF21*. Subsequently, Venn diagrams showed that the overlapping genes of the three algorithms were *PDLIM1*, *PARP14* and *SEL1L3* (**Figure 3F**).

### 3.5 Construction of a diagnostic model for AS

**Figure 4:**
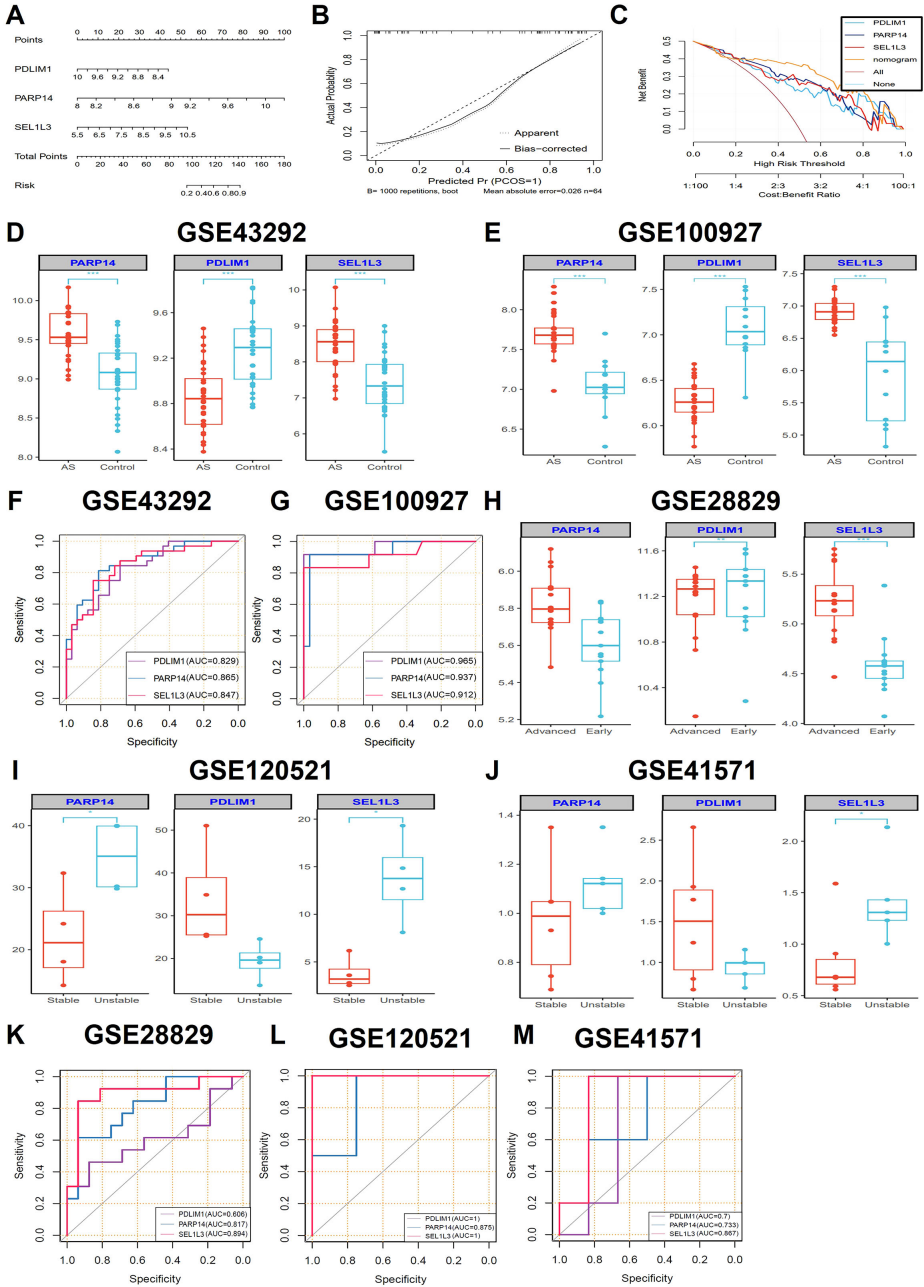
Construction of diagnostic nomogram and evaluation of diagnostic performance. **(A)** A diagnostic nomogram based on three characteristic genes was constructed. Each gene corresponded to a score and the total score of the three genes was used to predict the risk of AS (*n*=32 biologically replicated experiments.). **(B)** The calibration curves were constructed to evaluate the accuracy of the nomogram (*n*=32 biologically replicated experiments.). **(C)** DCA was performed to assess the net benefit of AS diagnostic decisions predicted by the nomograms (*n*=32 biologically replicated experiments.). **(D, E)** The expression comparisons of the three hub genes (PDLIM1, PARP14 and SEL1L3) in the GSE43292 (*n*=32 biologically replicated experiments.) and GSE100927 (AS groups, *n*=29 biologically replicated experiments; control groups, *n*=12 biologically replicated experiments.) training datasets. **(G, F)** ROC curves showing the performance of the three genes in the GSE43292 (*n*=32 biologically replicated experiments.) and GSE100927 (AS groups, *n*=29 biologically replicated experiments; control groups, *n*=12 biologically replicated experiments.) datasets. **(H-J)** The expression comparisons of the three hub genes (PDLIM1, PARP14 and SEL1L3) in the GSE28829 (advanced groups, *n*=16 biologically replicated experiments; early groups, *n*=13 biologically replicated experiments.), GSE120521 (*n*=4 biologically replicated experiments.) and GSE41571 (unstable groups, *n*=5 biologically replicated experiments; stable groups, *n*=6 biologically replicated experiments.) validation datasets. **(K-M)**. ROC curves showing the performance of the three genes in the GSE28829 (advanced groups, *n*=16 biologically replicated experiments; early groups, *n*=13 biologically replicated experiments.), GSE120521 (*n*=4 biologically replicated experiments.) and GSE41571 (unstable groups, *n*=5 biologically replicated experiments; stable groups, *n*=6 biologically replicated experiments.) validation datasets. Statistics performed by Independent sample T-test. *P < 0.05, **P < 0.01, ***P < 0.001.

We built a nomogram using the “rms” package, which integrates the diagnostic genes *PDLIM1*, *PARP14* and *SEL1L3* to predict the probability of AS. The predictive accuracy of these gene drive models was validated through calibration curve analysis (**Figure 4A**). The calibration curves demonstrated good agreement between the nomogram-predicted probabilities and the actual observed outcomes across the full risk spectrum (**Figure 4B**). Decision curve analysis (DCA) showed greater clinical utility of the nomogram (orange curve) compared to the full treatment strategy (brown curve) and individual biomarkers curves. The curves for *PDLIM1*, *PARP14*, and *SEL1L3* showed that patients could benefit from the nomogram model with a high-risk threshold of 0 to 1 (**Figure 4C**). In the training datasets GSE43292 and GSE100927, the expression of *PARP14* and *SEL1L3* were elevated in AS compared to the control group, whereas the expression of *PDLIM1* was reduced (**Figures 4D, E**). The diagnostic value of the characterized genes was further verified using ROC curves. In the GSE43292 and GSE100927 training sets, *PDLIM1* (AUC: 0.829; 0.965), *PARP14* (AUC: 0.865; 0.937), and *SEL1L3* (AUC: 0.847; 0.912) had high diagnostic value (**Figures 4F, G**). Notably, in the GSE28829 validation dataset, *PARP14* expression was elevated in advanced lesions compared to early lesions, although this difference did not reach statistical significance. *PDLIM1* expression was significantly lower in advanced lesions than in early lesions. Conversely, *SEL1L3* expression was significantly higher in advanced lesions compared to early lesions (**Figure 4H**). Furthermore, an analysis of the GSE120521 and GSE41571 validation datasets revealed that the *PARP14* and *SEL1L3* expression levels were elevated in unstable plaques compared to stable plaques, whereas *PDLIM1* expression was reduced (**Figures 4I, J**). In the GSE28829, GSE120521 and GSE41571 validation sets, *PDLIM1* (AUC: 0.606; 1; 0.7), *PARP14* (AUC: 0.817; 0.875; 0.733), and *SEL1L3* (AUC: 0.894; 1; 0.867) demonstrated significant diagnostic value (**Figures 4K–M**). Consequently, these three genes may serve as reliable diagnostic predictors for the AS.

### 3.6 The ssGSEA of characteristic genes

**Figure 5:**
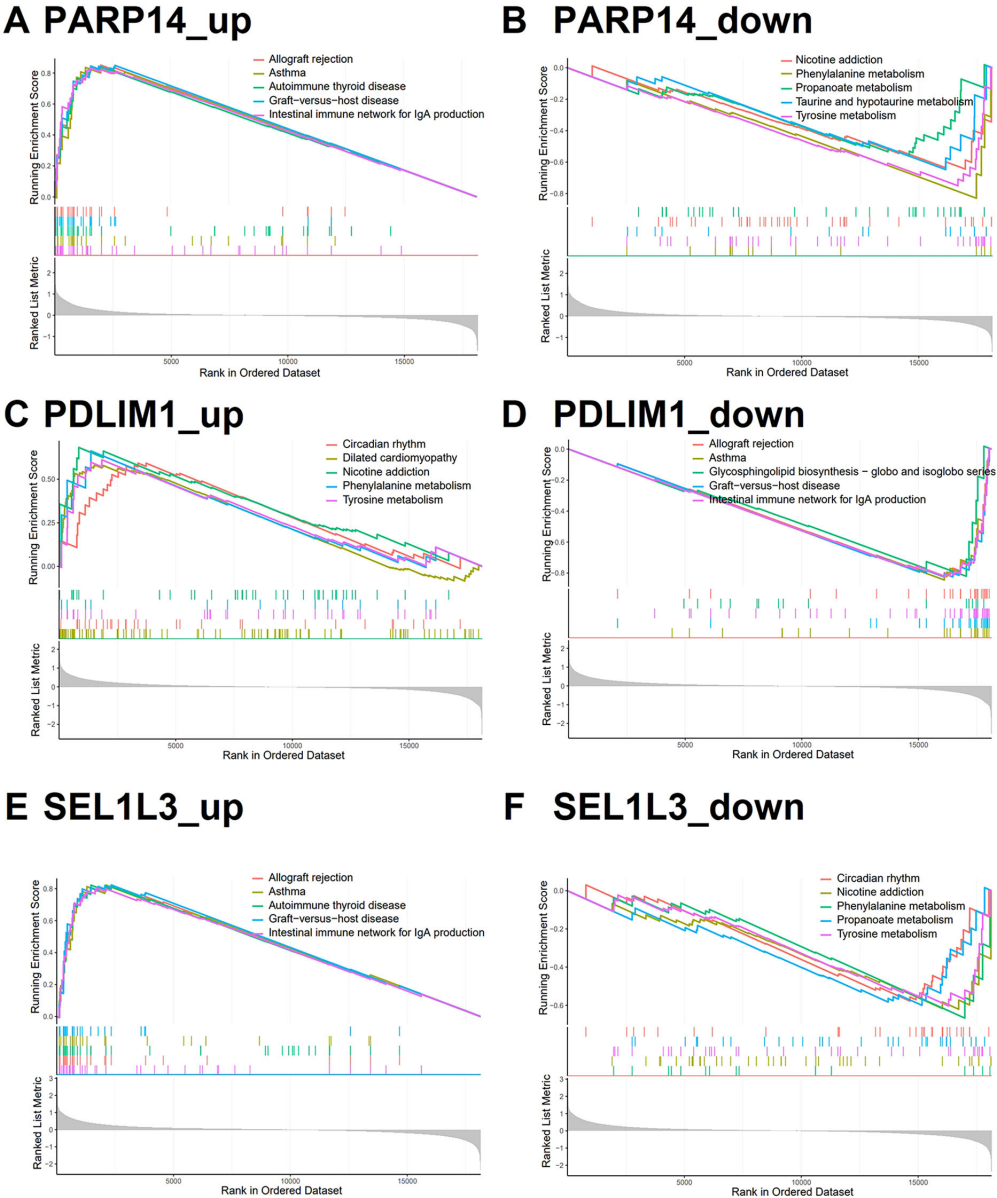
Characterized genes of single gene GSEA. **(A, D, E)** KEGG analysis of genes in the PDLIM1 low-expression group, PARP14 and SEL1L3 high-expression group using GSEA (top 5). **(B, C, F)** KEGG analysis of genes in the PDLIM1 high-expression group, PARP14 and SEL1L3 low-expression group using GSEA (top 5).

To elucidate the pathway activity patterns associated with the dynamics of *PDLIM1*, *PARP14*, and *SEL1L3* expression, we performed KEGG-based pathway activity analysis by ssGSEA. Samples stratified into the *PDLIM1* low-expression group, as well as *PARP14* and *SEL1L3* high-expression groups, exhibited significant enrichment of pathway activities associated with allogeneic rejection and immune responses (**Figures 5A, D, E**). Conversely, samples within the *PDLIM1* high expression group, *PARP14* and *SEL1L3* low expression groups predominantly exhibited enrichment in metabolic reactions (**Figures 5B, C, F**). These findings suggest that characterized genes may play an important role in the regulation of immune infiltration and metabolism processes.

### 3.7 Immune cell infiltration analysis in AS

**Figure 6:**
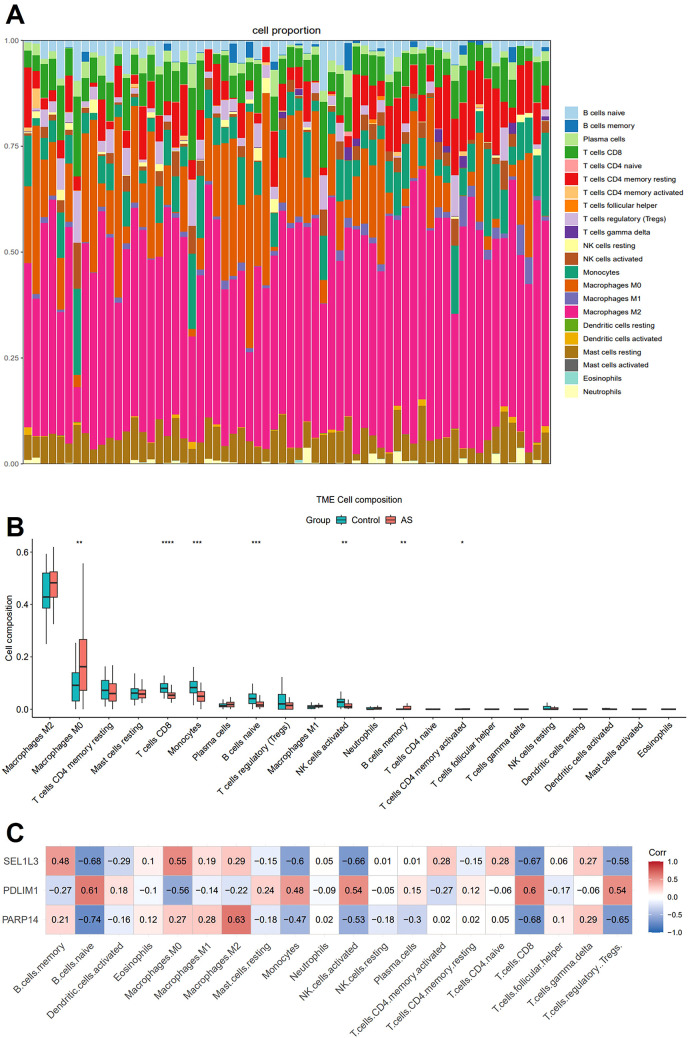
Immune cell infiltration analysis. **(A)** The stacked bar plot representing the proportions of different immune cells in each sample. **(B)** The boxplot depicting the comparison of the 22 types of immune cells between AS and controls. **(C)** The correlation heatmap showing the association of immune cells with the three characterized genes. Statistics performed by Independent sample T-test. *P < 0.05, **P < 0.01, ***P < 0.001, ****P < 0.0001, *n*=32 biologically replicated experiments.

We applied CIBERSORT with the LM22 signature matrix to estimate the proportions of 22 immune cell types in AS samples. **Figure 6A** illustrates the relative abundance of these immune cell populations across individual samples. Compared with the control group, the AS samples exhibited higher proportions of M0 macrophages, memory B cells, alongside decreased proportions of CD8^+^ T cells, monocytes, naive B cells and activated natural killer cells (**Figure 6B**). Subsequently, we conducted a Pearson correlation analysis to evaluate the relationship between the diagnostic genes and immune cells. Immune cells that exhibited a significant correlation with any of the characterized genes were included in the heat map. Interestingly, naive B cells, M0 macrophages, monocytes, activated natural killer cells, CD8^+^ T cells, and regulatory T cells constituted a substantial portion of AS plaque and exhibited significant correlations with the three diagnostic genes (**Figure 6C**). The correlation of immune cells with diagnostic genes may reflect either a driver role in AS pathogenesis or secondary responses to vascular inflammation.

### 3.8 The scRNA-seq reveals complex cellular features of AS lesions

**Figure 7:**
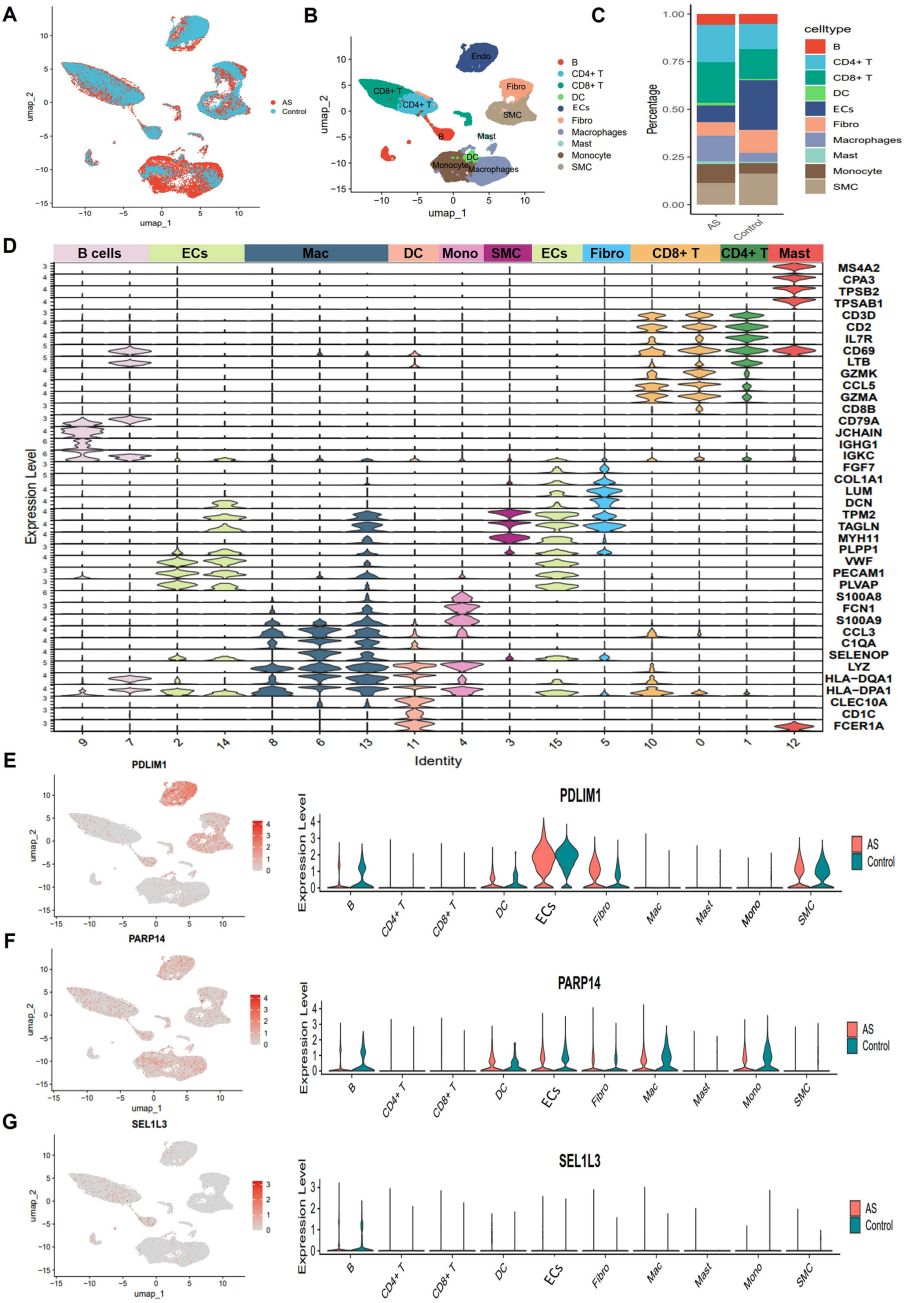
Single-cell sequencing reveals complex cellular features of AS lesions. **(A)** UMAP plot showing cell distribution from carotid and AS plaque samples. **(B)** Umap plot showing cellular composition in the AS microenvironment, colored according to cell types. **(C)** Proportion of major cell types between Control and AS groups. **(D)** Violin plot showing typical genealogical marker genes used to identify different cell types. **(E-G)** Uamp plot (left) and violin plot (right) showing the distribution and expression of PDLIM1, PARP14 and SEL1L3 in different cell clusters of AS.

To comprehensively characterize the cellular landscape of AS lesions, we collected the scRNA-seq dataset GSE159677, which comprises human carotid artery plaques. This dataset was generated using the 10x Genomics Chromium platform. Following stringent quality control and log-normalization, we performed an unbiased clustering of 46,278 high-quality cells. This analysis identified 13 subpopulations as shown in UMAP plot, which primarily included ECs, SMC, Fibro, CD4^+^ T cells, CD8^+^ T cells, B cells, monocytes, macrophages, and a minor presence of DC and mast cells (**Figures 7A, B**). Compared with the proportion of cell distribution in the control group, our analysis demonstrated a significant selective enrichment of T cells and macrophages in AS. The predominance of these immune cell populations in AS plaques supports the notion that the plaque-prone microenvironment drives the differential expansion of inflammatory cell lineages, rather than passive cell accumulation (**Figure 7C**). Cell types were annotated based on canonical markers such as *VWF* for ECs, *LUM* for Fibro, and *C1QA* for macrophages (**Figure 7D**). Subsequently, we determined the distribution of *PDLIM1*, *PARP14* and *SEL1L3* in ten integrated cell populations (**Figures 7E–G**).

### 3.9 Senescence status of individual cell populations

**Figure 8:**
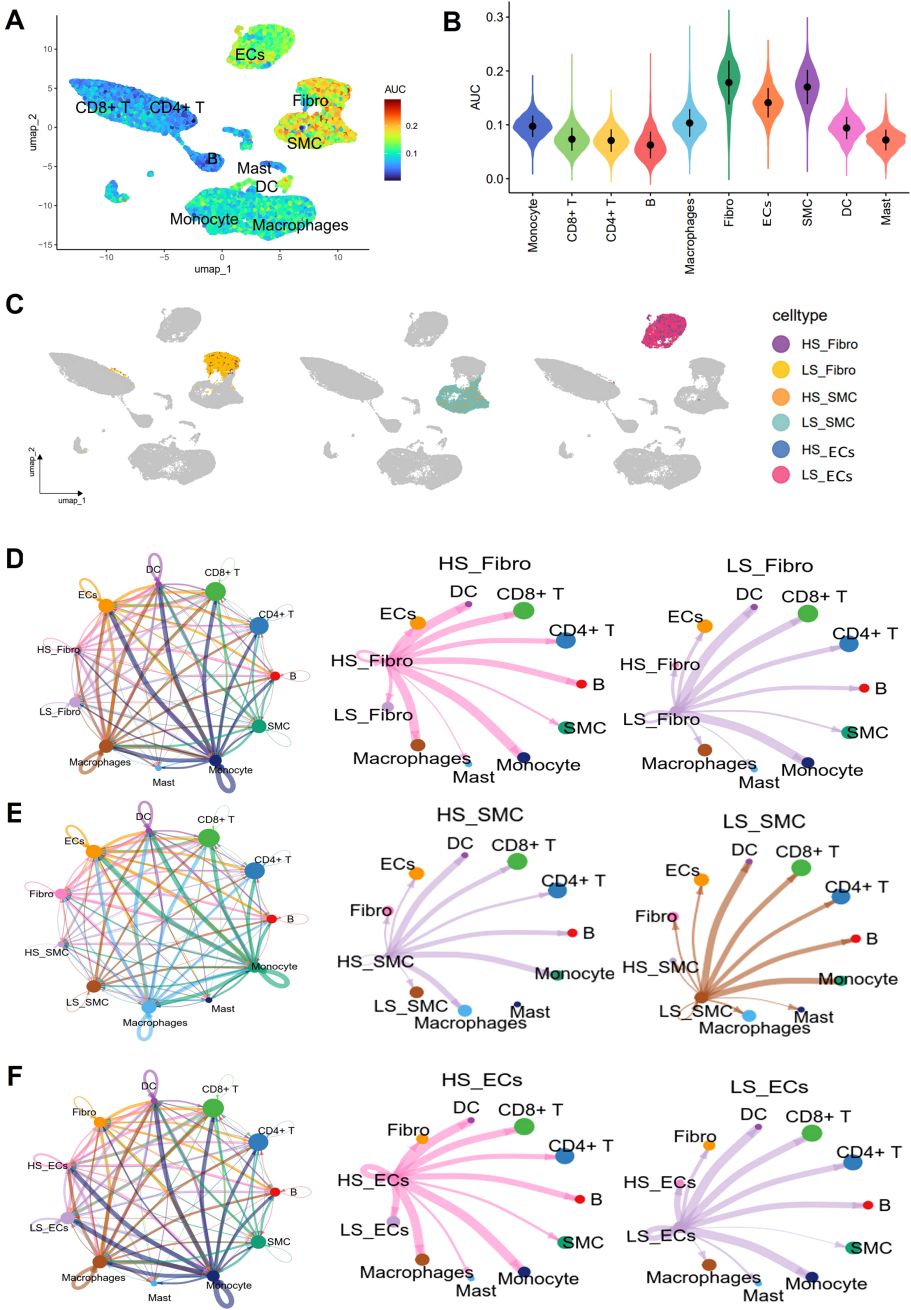
Cell interaction analysis using CellChat. **(A)** Umap plot showing senescence AUC scores for different cell types. **(B)** Violin plot showing differences in senescence AUC scores between different cell types. **(C)** Umap showing the distribution of high senescence (HS) and low senescence (LS) cells in Fibro, SMC and ECs in different cell clusters. Circle plots show the cellular interaction weights and number of interactions between HS cells, LS cells in **(D)** Fibro, **(E)** SMC, and **(F)** ECs, and other cell types in the AS microenvironment. Different colors in the circle diagram represent different cell types, and the edge width is proportional to the cell-cell interaction weights.

**Figure 9:**
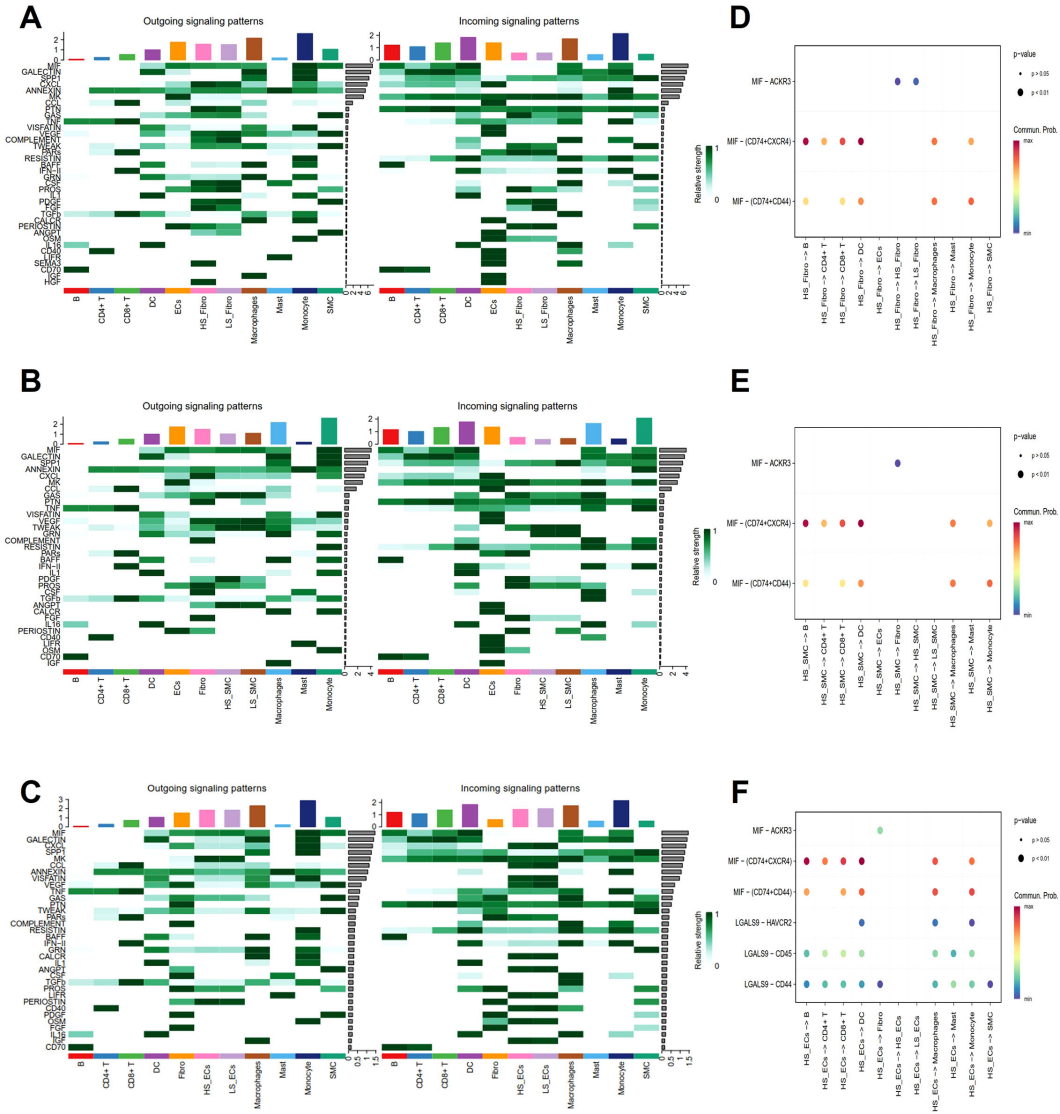
Analysis of cell-cell interaction signaling pathways. **(A-C)** Heatmaps show the outgoing (left) and incoming (right) signal intensities of each signaling pathway in different cell types. **(D-F)** Bubble plots show all the important ligand-receptor pairs that send signals from highly senescent cells in **(D)** Fibro, **(E)** SMC, and **(F)**. ECs to the other cell types. The dot colors and sizes in the bubble plots represent communication probability and p-values, with blue and red corresponding to minimum and maximum values, respectively.

As a measure of cellular senescence, we employed a well-established set of SRGs (FRIDMAN. SENESCENCE. UP, **Supplementary Table 1**). Subsequent AUCell scoring of these cells based on FRIDMAN. SENESCENCE. UP set, revealed that Fibro, ECs, and SMC exhibited higher scores compared to other cell populations, including macrophages, and T cells (**Figures 8A, B**). To further explore the overall communication between senescent cells and other cell populations in the plaques, we categorized Fibro, ECs, and SMC into HS and LS cells, respectively (**Figure 8C**). In order to systematically map the dynamics of intercellular communication, we performed a cell-cell interaction analysis using CellChat. The CellChat analysis indicated that HS cells established significantly more ligand-receptor interactions in the AS microenvironment compared to LS cells. Notably, HS-Fibro, HS-SMC and HS-ECs exhibited stronger communication primarily with macrophages, although B cells and SMC were also involved (**Figures 8D–F**). Specifically, it was computationally inferred that HS cells release the cytokines macrophage migration inhibitory factor (*MIF*) and β-galactoside-binding-lectins (*GALECTIN*, **Figures 9A–C**), utilizing six main pathways, including *MIF*-(*CD74^+^CXCR4*), *MIF-(CD74^+^CD44)*, *MIF-ACKR3*, *Galectin9 (LGALS9)-CD45*, *LGALS9-HAVCR2*, and *LGALS9-CD44* (**Figures 9D–F**). Thus, our data suggest that senescent cells in AS lesions exert potent immunomodulatory effects, primarily by activating macrophage-driven inflammatory responses through *MIF*- and galectin-dependent pathways.

### 3.10 Single cell analyses reveal unique cardiac macrophage subsets

**Figure 10:**
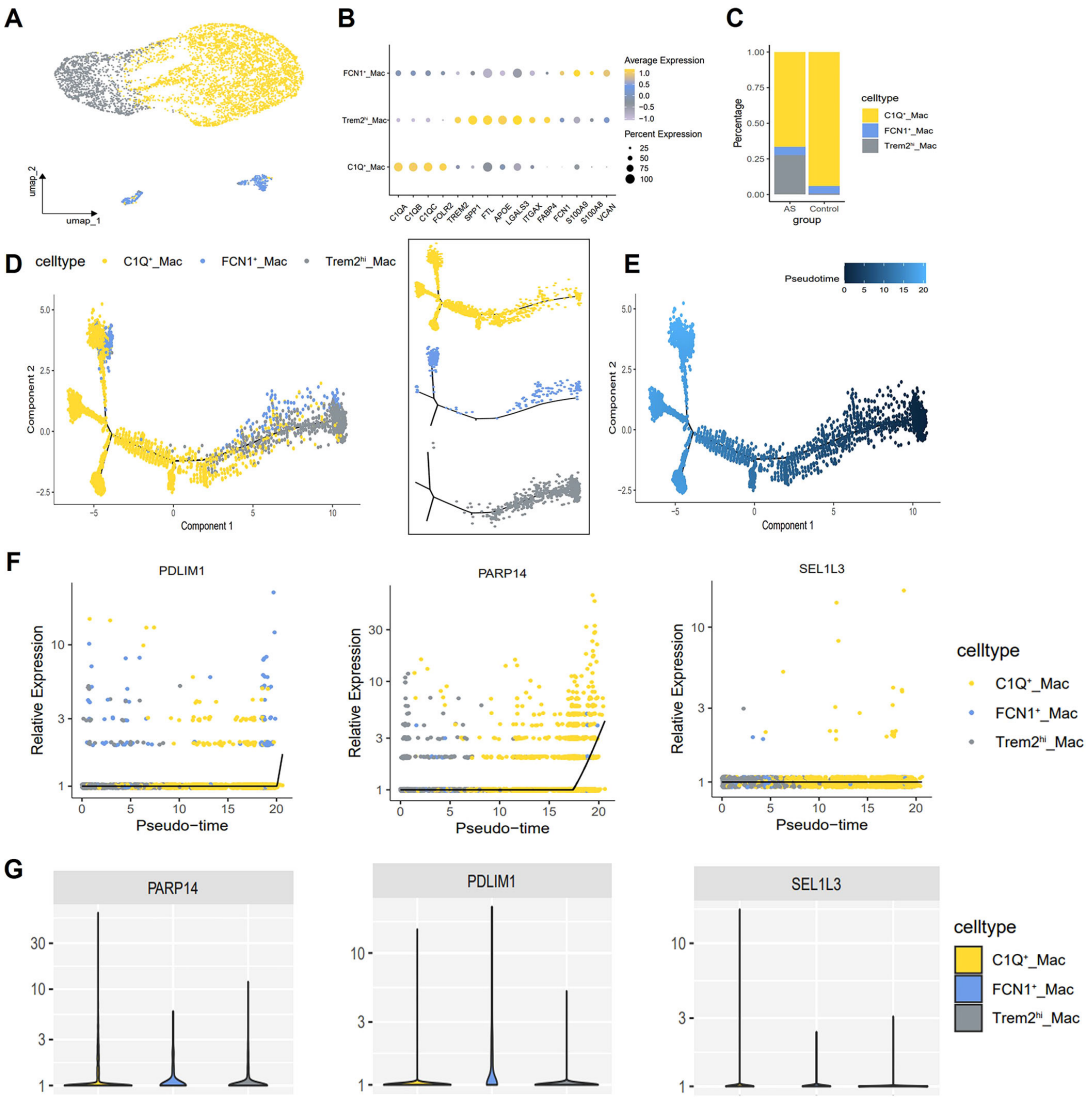
Macrophage single-cell mapping and trajectory analysis. **(A)** Umap showing the clustering and annotation of macrophages. **(B)** Bubble plot showing canonical lineage marker genes used to identify distinctive cell types. **(C)** Bar plots showing the composition ratio of each macrophage cluster. **(D, E)** Trajectory map indicating the developmental correlations of the 3 clusters. **(F)** Diagnostic genes expression changes over pseudotime. x-axis indicates cells analyzed by trajectory analysis, y-axis represents the relative expression of genes. **(G)** Violin plot showing the expression of diagnostic genes in the 3 macrophage subtypes.

Subsequently, macrophages were extracted from the integrated dataset and subjected to reclustering using standard Seurat procedures. A total of 5,295 high-confidence macrophages and 21,027 genes were retained for downstream analysis. Further annotation identified three subtypes: TREM2^hi^ macrophages, C1Q^+^ macrophages and FCN1^+^ macrophages (**Figure 10A**). The C1Q^+^ macrophages exhibited high expression levels of complement genes *C1QA*, *C1QB*, *C1QC*, as well as M2-like macrophage related genes (*FOLR2*), categorizing them as AS-resident macrophages, which functionally resemble M2 macrophages (30). Trem2^hi^ macrophages were characterized by elevated expression of lipid accumulation, including *TREM2* and *FABP4*, and were also enriched in *LGALS3*, *ITGAX* (31, 32). GO and KEGG analysis showed that Trem2^hi^ macrophages are involved in lipid metabolism, cholesterol efflux and lysosomal functions (33, 34). These Trem2^hi^ macrophages appear to be foam cells, but they did not produce inflammatory cytokines or chemokines associated with AS. In contrast, FCN1^+^ macrophages highly express inflammatory monocyte-associated genes, such as *S100A9*, *S100A8*, and *VCAN*, suggesting a proinflammatory role for this subset in AS (**Figure 10B**) (30). Notably, C1Q^+^ macrophages were significantly reduced and Trem2^hi^ macrophages were significantly increased in the AS group compared to the control group (**Figure 10C**).

### 3.11 Single cell trajectories reveal developmental relationships

To further investigate the relationship between different macrophages populations, we employed the Monocle2 package for pseudotime analysis and utilized the DDRTree algorithm for dimensionality reduction. Clusters defined by Seurat were superimposed on a pseudotime trajectory generated by the Monocle algorithm, which revealed that TREM2^hi^ macrophages and FCN1^+^ macrophages occupied a separate branch of the trajectory. In contrast, C1Q^+^ macrophages spanned each branch of the trajectory while maintaining transcriptional continuity. Transcription of TREM2^hi^/FCN1^+^ macrophage clusters gradually transitioned to a C1Q-enriched state (**Figures 10D, E**). Subsequently, Gene expression was plotted in Monocle2 as a function of pseudotime to identify genes driving macrophage state transitions. We identified *PARP14*, *PDLIM1* and *SEL1L3* as genes exhibiting increased expression in C1Q^+^ macrophages (**Figures 10F, G**).

### 3.12 Biomarker expression in AS mice

**Figure 11:**
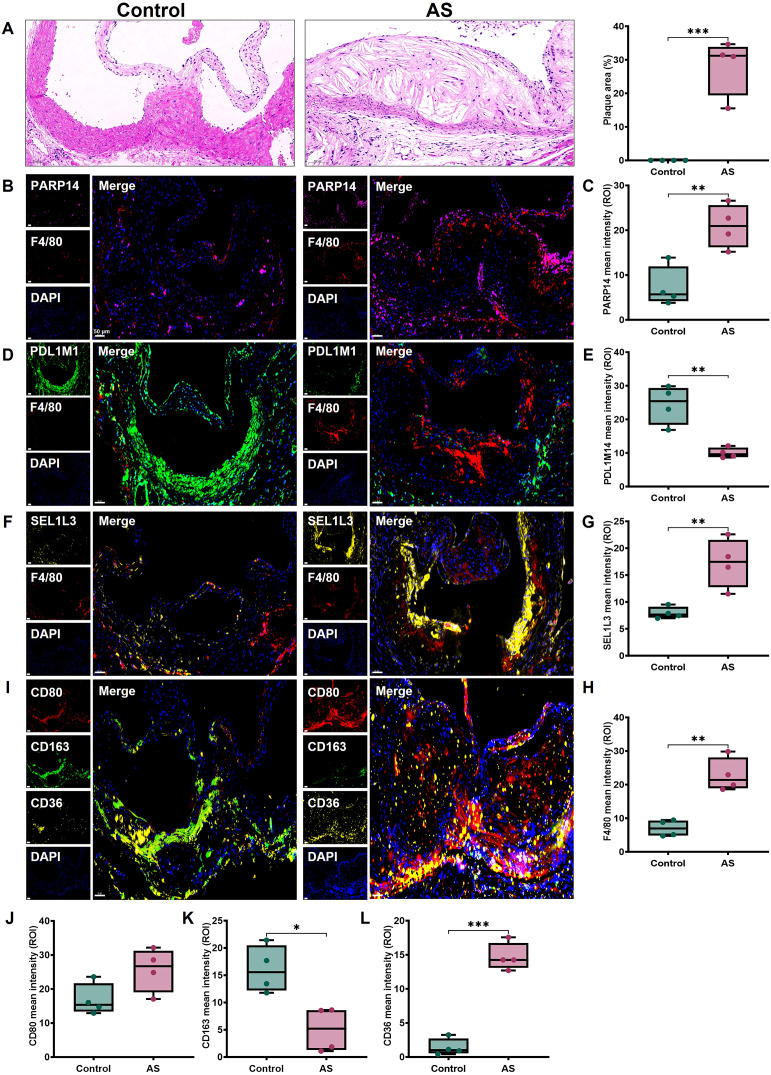
Expression of biomarkers in mice AS tissues. **(A)** Representative images of aortic root sections stained with HE and quantitative analysis of plaque area. Representative images and protein levels of immunofluorescence staining for **(B, C)** PARP14, **(D, E)** PDLIM1, **(F, G)** SEL1L3, **(B, D, F, H)** F4/80, **(I, J)** CD80, **(I, K)** CD163, **(I, L)** CD36 in mice aortic root, respectively. Scale bar, 50 μm. Statistics performed by independent sample T-test. *P < 0.05, **P < 0.01, ***P < 0.001, *n*=4 biologically replicated experiments, *n*=3 technically repeated experiments.

HE staining analysis showed significant plaque accumulation in the aortic sinus in the AS group, characterized by lipid-rich areas and cholesterol crystal-like structures with inflammatory cell infiltration (**Figure 11A**). We measured PDLIM1, PARP14 and SEL1L3 protein expression in mice aortic valves. Immunofluorescence staining showed that PDLIM1 expression was significantly down-regulated, while PARP14 and SEL1L3 expression were significantly up-regulated in the aortic root in the AS groups compared with the control groups (**Figures 11B–G**). And macrophages labelled by F4/80 also showed a significant up-regulation in the AS group (**Figures 11B, D, F, H**). We further labelled M1 macrophages with CD80, M2 macrophages with CD163 and foam cells with CD36, which showed that CD163 expression was significantly reduced, CD80 and CD36 expression was significantly upregulated in the AS group compared with the control group (**Figures 11I–L**). These findings align with our previous analysis. The above results suggest that the dysregulation of the M1/M2 macrophage ratio, along with a substantial proliferation of foam cells, contributes to the progression of AS. This process may be facilitated by the stimulation of macrophage transformation by SRGs such as *PDLIM1*, *PARP14*, and *SEL1L3*.

## 4 Discussion

Early detection, prevention and intervention of AS are key to reducing morbidity and mortality, as well as alleviating the enormous socioeconomic burden of atherosclerotic CVD (35, 36). Current clinical strategies rely on imaging modalities (such as coronary artery calcium scoring, carotid ultrasound) and lipid analysis for risk stratification. However, traditional biomarkers, such as LDL do not fully predict residual risk in statin-treated patients —— risk of persistent cardiovascular events caused by non-lipid mechanisms such as residual inflammatory risk, thrombotic tendency, and metabolic derangements (37). Imaging techniques such as computed tomography angiography lack sensitivity in identifying vulnerable plaques with high lipid content or intraplaque hemorrhage (38). Advanced age constitutes a significant risk factor for AS, driven by both immune senescence and endothelial dysfunction. Aging is characterized by impaired immune surveillance and chronic low-grade inflammation (39). Key molecular mechanisms include the senescence-associated secretory phenotype, where senescent ECs, macrophages, and VSMC release pro-inflammatory cytokines (such as *IL-6*, *IL-1β*), matrix metalloproteinases, and reactive oxygen species. These factors collectively contribute to the destabilization of atherosclerotic plaques and expedite vascular remodeling (40, 41). In the present study, our findings confirmed that the SRGs poly (ADP-ribose) polymerase family member 14 (*PARP14*), SEL1L family member 3 (*SEL1L3*) and PDZ and LIM domain 1 (*PDLIM1*) are diagnostic genes of AS. These genes exhibit robust diagnostic capabilities, effectively distinguishing between early and advanced AS lesions, as well as between stable and unstable plaques. In addition, our results suggest that *PDLIM1*, *PARP14* and *SEL1L3* may drive macrophage transformation and promote the progression of AS lesions.

In this study, we conducted a comprehensive bioinformatics analysis that identified 234 AS key genes between the AS and control groups. Functional enrichment analysis of these genes revealed significant associations with terms related to immune cell engagement, immune activation, and inflammation. These findings suggest a strong link between AS and immune and inflammatory processes, which is consistent with current views (36). Importantly, we screened 89 SRGs from 234 key genes for AS. In order to prioritize robust biomarkers, we applied 3 machine learning models (LASSO, RF, SVM) with different feature selection principles to further identify key features of AS. Extracting the intersection of markers from the combination of the three algorithms can further reduce the number of markers and thus improve the specificity and sensitivity of the signature. Ultimately, *PDLIM1*, *PARP14* and *SEL1L3* were used as diagnostic biomarkers for AS. Further ROC analysis of these diagnostic genes revealed that *PDLIM1*, *PARP14* and *SEL1L3* could effectively distinguish between individuals with AS and those without the condition. Their AUC values were consistently greater than 0.7 in both the training and validation cohorts, suggesting their strong discriminatory ability. These results suggest that *PDLIM1*, *PARP14* and *SEL1L3* have great potential for clinical applications in disease prediction, early diagnosis and disease stratification in AS.

Given the important role of immunity in AS, we sought to explore the relationship between diagnostic genes and immune cells in AS. It was found that *PDLIM1*, *PARP14* and *SEL1L3* showed different degrees of correlation with immune cells such as B cells, macrophages, monocytes and T cells. Very importantly, we found differences in the expression of *PDLIM1*, *PARP14* and *SEL1L3* between the two groups of macrophages by analyzing scRNA-seq data. The scRNA-seq technique reveals significant expansion of senescent EC and VSMC by resolving cellular heterogeneity of AS, confirming the pathological mechanism of vascular senescence driving AS progression at the cellular dynamic level (42). It is now widely recognized that vascular senescence and AS are mutually reinforcing processes, sharing common risk factors. Surprisingly, we found that senescent vascular cells (EC, SMC, and Fibro) exhibited stronger communication with macrophages via *MIF* and lectins by cell communication analysis. These interactions not only locally recruit more monocyte-macrophages to migrate to the subendothelium, where they undergo phenotypic changes due to senescence-associated secretions and transform into foam cells, but also induce plaque formation by SMCs and inflammatory cells through phenotypic changes in exosome secretion (42). This mechanistic insight precisely accounts for the observed increase in foam cells within the AS group. Researchers have identified the proinflammatory cytokine *MIF* as a major mediator of AS. Plasma *MIF* levels are associated with arterial stiffness, which serves as a marker of vascular aging. Preclinical studies have shown that blocking *MIF* leads to regression of AS plaques (43, 44). In addition, HS cells have been found to release lectins, a carbohydrate-binding protein known to support immune cell migration and vascular program reprogramming. Thus, our study suggests that senescent vascular cells and macrophages play a role in the progression of AS lesions.

Senescent cells generally engage the immune system to facilitate their clearance from tissues. The accumulation of senescent cells in AS is thought to result from deficient immune surveillance, and immunostimulants have been shown to increase the removal of these cells (45). Empirical evidence supports the notion that aging promotes proinflammatory changes in monocytes/macrophages associated with AS. In addition, altered adhesion molecules on aged EC are expected to promote macrophage migration and activation within plaques with aging (46). The accumulation of senescent cells in AS is hypothesized to result from impaired immune surveillance, with immunostimulants demonstrated to enhance the clearance of these cells. There is growing evidence that macrophages may play a driving role in all stages of AS, from the formation of “fatty streaks” to the development of complex plaques. Senescent foamy macrophages are implicated in the initiation of early AS and complex advanced lesions. In addition, the removal of senescent macrophages has been shown to stabilize AS by reducing inflammatory cytokines, monocyte recruitment factors and plaque destabilization-related matrix metalloproteases (47–50). For a considerable period, plaques have been believed to encompass multiple phenotypically diverse macrophage subpopulations (27, 51). The advancement of scRNA-seq has transcended the conventional classification of macrophages into M1 and M2 types. Instead, it facilitates an unbiased characterization of cellular heterogeneity and enables the identification of cellular identities through labeling strategies. Furthermore, it significantly enhances the ability to uncover previously unrecognized cell populations or functional states associated with diseases, along with their specific markers and the molecular regulators that underpin them (33, 52). In this study, we conducted an in-depth analysis of the macrophage population to discover three major macrophage populations in AS plaques, including TREM2^hi^ macrophages, C1Q^+^ macrophages and FCN1^+^ macrophages. We further characterized their gene expression profiles. Notably, the C1Q^+^ macrophages subset is characterized by the expression of genes encoding the complement C1q chains, which play crucial roles in atheroprotective functions of macrophages, including the reversal of cholesterol transport, the attenuation of inflammation, and the facilitation of cellular clearance (53). The TREM2^hi^ macrophages subpopulation, a new macrophage lineage also previously reported in the literature (53). *TREM2* expression in antigen-presenting cells has been previously described in human disease, where it is hypothesized to have a negative correlation with plaque stability (54). It was suggested that *TERM2*-expressing myeloid cells may arise in response to microenvironmental stressors that are commonly associated with neurological disorders and AS, such as localized inflammation, altered lipid metabolism, and protein misfolding. Pseudotime analysis revealed that TREM2^hi^ macrophages and FCN1^+^ macrophages transformed to C1Q^+^ macrophages over time. C1Q^+^ macrophages predominate in advanced plaques, where they facilitate cholesterol efflux and mitigate inflammation. Plaque regression has been reported to be dependent on monocyte recruitment and differentiation to anti-inflammatory rather than pro-inflammatory macrophages, which may indicate an important influence of macrophage phenotype in the plaque microenvironment (55).

A previous study showed that *PDLIM1* knockdown reversed miR-150 ablation-induced suppression of expression and macrophage infiltration (56). The expression level of *PDLIM1* was significantly downregulated in advanced AS compared to early AS, and models constructed using *PDLIM1* have a good diagnostic performance for AS (57). *PARP14*, a member of the *PARP* family, has been implicated as a potential molecular switch for macrophage activation, cross-regulating macrophage M1 and M2 polarization in the context of AS (58). The *PARP* family (especially *PARP1* and *PARP14*) is involved in lipid metabolism and regulates lipid metabolism and homeostasis *in vivo* through transcription factors that play a central role in AS development (59). It was shown that *PARP14* inhibits *STAT1* phosphorylation and nuclear translocation via ADP-ribosylation, thereby blocking the expression of pro-inflammatory genes. Meanwhile, *PARP14* catalyzes ADP-ribosylation of HDAC2/3 in response to *IL-4* stimulation and rescinds its transcriptional repression of *STAT6*, thereby promoting anti-inflammatory M2-type macrophage polarization. This dynamic equilibrium mechanism explains the central role of *PARP14* in regulating the inflammatory response (60, 61). *SEL1L3*, a member of the SEL1L (Sel-1 Suppressor of Lin-12-Like) family, is situated within the endoplasmic reticulum (ER) and plays a pivotal role in enabling ER-associated degradation. This degradation process is triggered by ER stress, which promotes the breakdown of misfolded proteins. ER stress drives intraplaque inflammation and necrotic core formation via macrophage lipid accumulation and *NLRP3* inflammasome activation (62). *SEL1L3* was found to be highly expressed in AS tissues, which is consistent with the results of our analysis, but the exact mechanism has not been explored (63). We suggest that these SRGs may drive macrophage transformation and drive the progression of AS lesions. This evidence offers novel insights into the pathogenesis of AS and its associated immune mechanisms, which could inform strategies for targeted prevention, disease monitoring, and therapeutic intervention.

The early detection and diagnosis of AS are particularly important for the prognosis of the disease. Carotid intima-media thickness, ankle-brachial index, and pulse wave velocity are commonly used to predict early AS changes. However, there remains a lack of satisfactory biomarkers for effective screening and early diagnosis of AS (4, 64). Vascular senescence has a significant impact on AS lesions. Deciphering vascular senescence is the cornerstone of developing new therapies against AS targeting lipid metabolism and inflammation. Primary prevention of AS is crucial for the management of atherosclerotic CVD. In our study, we found that higher levels of *PARP14* and *SEL1L3* might be associated with more severe disease. The integration of *PDLIM1, PARP14* and *SEL1L3* detection with established diagnostic modalities could potentially assist clinicians in identifying individuals at elevated risk for AS and enabling early-stage diagnosis, demonstrating considerable promise for clinical application in disease surveillance and risk stratification. Our study is subject to several noteworthy limitations that warrant acknowledgment. First, while the use of public scRNA-seq datasets provided valuable exploratory insights, this approach inherently restricted our access to extensive clinical metadata, such as patient age, gender distribution, and comorbidities. Secondly, while this study has conducted preliminary screening of key marker genes for AS, subsequent validation through *in vivo* and *in vitro* experimental models remains imperative. Finally, our analytical approach is subject to technical limitations inherent in public sequencing datasets, including potential batch effects between different experimental platforms, variability in single-cell capture efficiencies, and relatively limited sample sizes. These factors may affect the generalizability of our findings, necessitating a cautious interpretation of the results.

## 5 Conclusions

In conclusion, our study reveals that immune mechanism-mediated SRGs *PDLIM1*, *PARP14*, and *SEL1L3* may collectively participate in the mechanism of AS formation. The dynamic interaction between the vascular senescence microenvironment and immune cells emerges as a critical pathological link facilitating AS progression. Specific up-regulation patterns of *PARP14* and *SEL1L3* in diseased tissues provide potential research directions for the development of targeted prevention strategies, precision diagnostic biomarkers, disease stratification systems and novel immunotherapies for AS.

## Data Availability

The datasets presented in this study can be found in online repositories. The names of the repository/repositories and accession number(s) can be found below: https://www.ncbi.nlm.nih.gov/geo/, GSE43292 https://www.ncbi.nlm.nih.gov/geo/, GSE28829 https://www.ncbi.nlm.nih.gov/geo/, GSE100927 https://www.ncbi.nlm.nih.gov/geo/, GSE120521 https://www.ncbi.nlm.nih.gov/geo/, GSE41571 https://www.ncbi.nlm.nih.gov/geo/, GSE159677.
